# Do Baseline P-Values Follow a Uniform Distribution in Randomised Trials?

**DOI:** 10.1371/journal.pone.0076010

**Published:** 2013-10-01

**Authors:** Martin Bland

**Affiliations:** Department of Health Sciences, University of York, York, North Yorkshire, United Kingdom; University of New South Wales, Australia

## Abstract

**Background:**

The theory has been put forward that if a null hypothesis is true, P-values should follow a Uniform distribution. This can be used to check the validity of randomisation.

**Method:**

The theory was tested by simulation for two sample t tests for data from a Normal distribution and a Lognormal distribution, for two sample t tests which are not independent, and for chi-squared and Fisher’s exact test using small and using large samples.

**Results:**

For the two sample t test with Normal data the distribution of P-values was very close to the Uniform. When using Lognormal data this was no longer true, and the distribution had a pronounced mode. For correlated tests, even using data from a Normal distribution, the distribution of P-values varied from simulation run to simulation run, but did not look close to Uniform in any realisation. For binary data in a small sample, only a few probabilities were possible and distribution was very uneven. With a sample of two groups of 1,000 observations, there was great unevenness in the histogram and a poor fit to the Uniform.

**Conclusions:**

The notion that P-values for comparisons of groups using baseline data in randomised clinical trials should follow a Uniform distribution if the randomisation is valid has been found to be true only in the context of independent variables which follow a Normal distribution, not for Lognormal data, correlated variables, or binary data using either chi-squared or Fisher’s exact tests. This should not be used as a check for valid randomisation.

## Introduction

I have seen the theory put forward that if a null hypothesis is true, P-values should follow a Uniform distribution. I came across this idea in two different contexts, neither published.

The first context was when reviewing a paper proposing methods to investigate the quality of published clinical trials. It was suggested as an indicator of valid randomisation in, and valid conduct of, a clinical trial. A reviewer could look at the distribution of the P-value in comparisons of baseline data between the randomised groups. All the null hypotheses should be true, so this distribution should be Uniform if participants were randomised fairly. Many triallists would object that such tests are meaningless because if all null hypotheses are known to be true then any positive significance tests must be type 1 errors. From this viewpoint, the presence of such tests is an indicator of bad reporting and low quality. However, reviewers can do the tests themselves, given published means and standard deviations or counts. A further objection is that such tests within a study are not independent. For example, triallists might report variables such as height and weight, or diastolic and systolic blood pressure. These variables would be correlated. Would the tests have to be independent for this idea to work?

The second context was in an analysis, planned by a colleague, as an indicator of valid randomisation in a meta-analysis of clinical trials. The research hypothesis was that randomisation is sometimes subverted by recruiters. They might do this, for example, by not recruiting a potential participant to a trial if the next treatment, which the recruiter knows or guesses, would be unsuitable in the recruiter’s opinion. Recruiters may do this because they want to favour the intervention by putting less sick or fitter people in intervention group, they may want to protect frailer people from the intervention, or they may want to make sure that sicker people get it. Motivations may be venal or benign, but either would invalidate the trial. An indication almost always reported as a baseline measure in clinical trials is age. A reviewer could test ages between randomised groups, and would expect to get a Uniform distribution for the P-values if the randomisations were all unbiased. Here the reviewer, rather than the trial researchers, would be doing the tests on the baseline ages. The tests would be independent, because each one is for a different trial.

The same idea has been used by others in a less developed way, looking at the proportion of baseline tests which have P<0.05 and arguing that this should be 5%. Schulz et al. [1] reported that, in 125 published trials in which hypothesis tests had been used to compare baseline characteristics, only 2% of 1076 test results were statistically significant, lower than the expected rate of 5%, a significant discrepancy (P<0.001).

If P-values do not appear to fit a Uniform distribution, is it valid to conclude that the randomisation has been compromised? Theory certainly suggests that P-values should follow a Uniform distribution if the null hypothesis is true. However, two things could upset this:

1. Tests might not be valid. If a t test is carried out for data which are from a highly skewed distribution, then this is repeated following a log transformation to a Normal distribution, a different P-value is obtained. In my experience, this is usually smaller. So if tests are not valid, would the P-values follow a Normal distribution?
2. If tests are not independent, the same test is, in effect, repeated several times. How can the P-values follow a Uniform distribution?

In this paper I test these ideas by simulation.

## Materials and Methods

The simulations were done using Stata 12 (Stata Corp., College Station, Texas) as follows:

### 1. The two sample t test with Normal data

The basic simulation generated two groups of 10 observations from a Standard Normal distribution. The two sample t test was used to test the true null hypothesis that the means were the same in the two populations. This was repeated 10,000 times and the distribution of the resulting P-values displayed as a histogram.

### 2. The two sample t test with highly skewed data

The basic simulation generated two groups of 10 observations from a Standard Normal distribution and then exponentiated these to give a variable which had a Lognormal, highly positively skewed distribution. The two sample t test was used to test the true null hypothesis that the means were the same in the two populations. This was repeated 10,000 times and the distribution of the resulting P-values displayed as a histogram.

### 3. The two sample t test with Normal data where the tests are correlated

Two groups of 10 observations from a Standard Normal distribution were generated. For each basic simulation, a second, correlated variable was generated by adding another Standard Normal variate to the first. This was repeated 10,000 times, as above. The variables thus generated had an expected correlation of 0.5 between each pair.

### 4. Binary data in a small sample

To compare two groups for a binary variable, such as gender, a chi-squared test or Fisher’s exact test would be used. As in the previous simulations, two samples of size 10 were generated, this time with a binary outcome variable with probability 0.5 of being 0 and 0.5 of being 1. The uncorrected chi-squared test and Fisher’s exact tests were carried out. This was repeated 10,000 times.

### 5. Binary data in a large sample

The chi-squared test would not usually be regarded as valid for a two way table with only 20 observations, though Fisher’s exact test should be. The simulation was therefore repeated using two samples of size 1,000.

## Results

### 1. The two sample t test with Normal data

**Figure 1:**
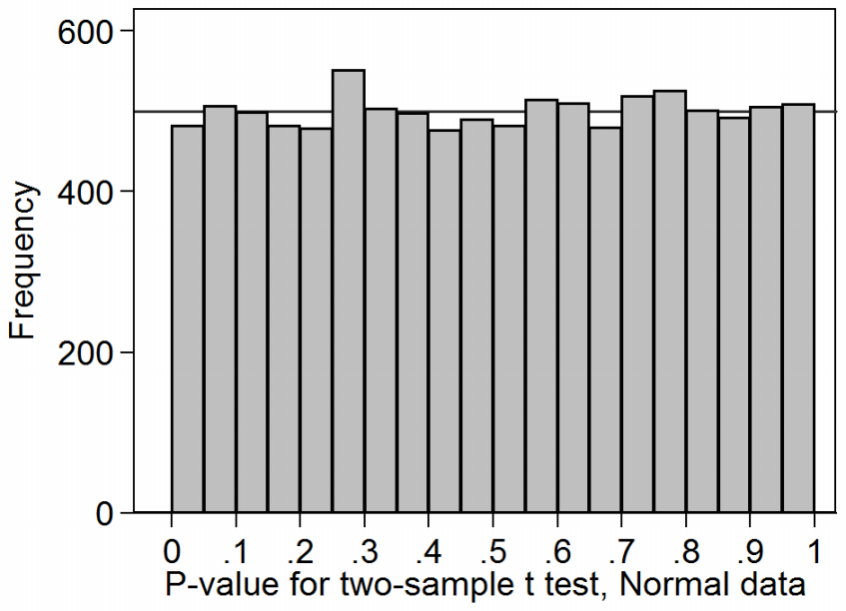
Distribution of P-values for 10,000 two sample t tests for Normal data. Means were compared between two groups of 10 observations from a Standard Normal distribution.

Figure 1 shows the distribution of P-values for 10,000 two sample t test comparing means in two groups of 10 observations from a Standard Normal distribution. If the P-values followed a Uniform distribution, all the frequencies should be close to 500. This is indeed the case and the P-values do appear to follow a Uniform distribution.

### 2. The two sample t test with highly skewed data

**Figure 2:**
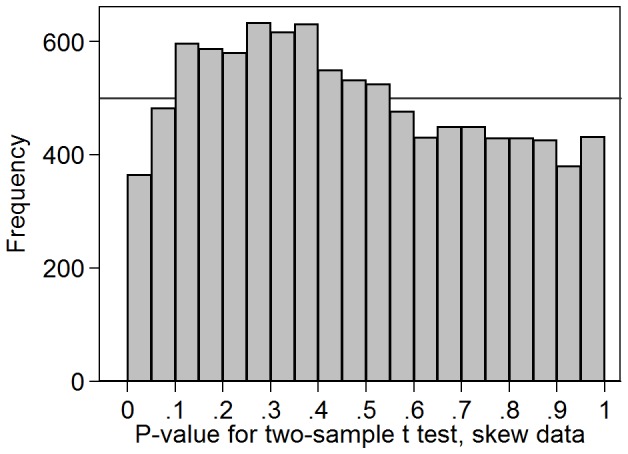
Distribution of P-values for 10,000 two sample t tests for Lognormal data. Means were compared between two groups of 10 observations from a Lognormal distribution.

Figure 2 shows the results of the simulation for 10,000 two sample t tests using data from a Lognormal distribution. The distribution of P-values is not Uniform, with an excess of P-values in the region 0.1 to 0.4 and deficits in the regions 0.0 to 0.05 and 0.06 to 1.0. The distribution has a distinct mode and fewer values less than 0.05, indicating conventional statistical significance, than was found with the Normal data.

### 3. The two sample t test with Normal data where the tests are correlated

**Figure 3:**
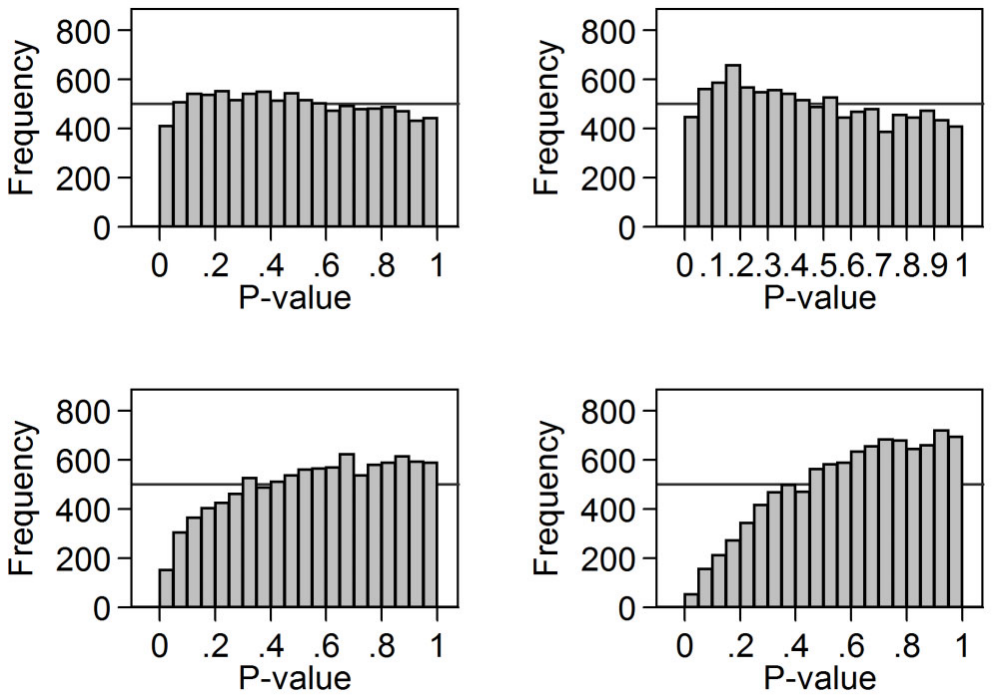
P-values from four realisations of 10,000 correlated t tests for Normal data. Means were compared between two groups of 10 observations from a Standard Normal distribution where each test used variables with correlation 0.5 with the other variables.

Figure 3 shows the results of valid t tests which are not independent, using correlated data. As the 10,000 repetitions are correlated, the results will depend on the values of the starting random observations. For this reason, four realisations of 10,000 replications are shown. The shapes of the distributions vary, but none are close to a Uniform distribution.

### 4. Binary data in a small sample

**Figure 4:**
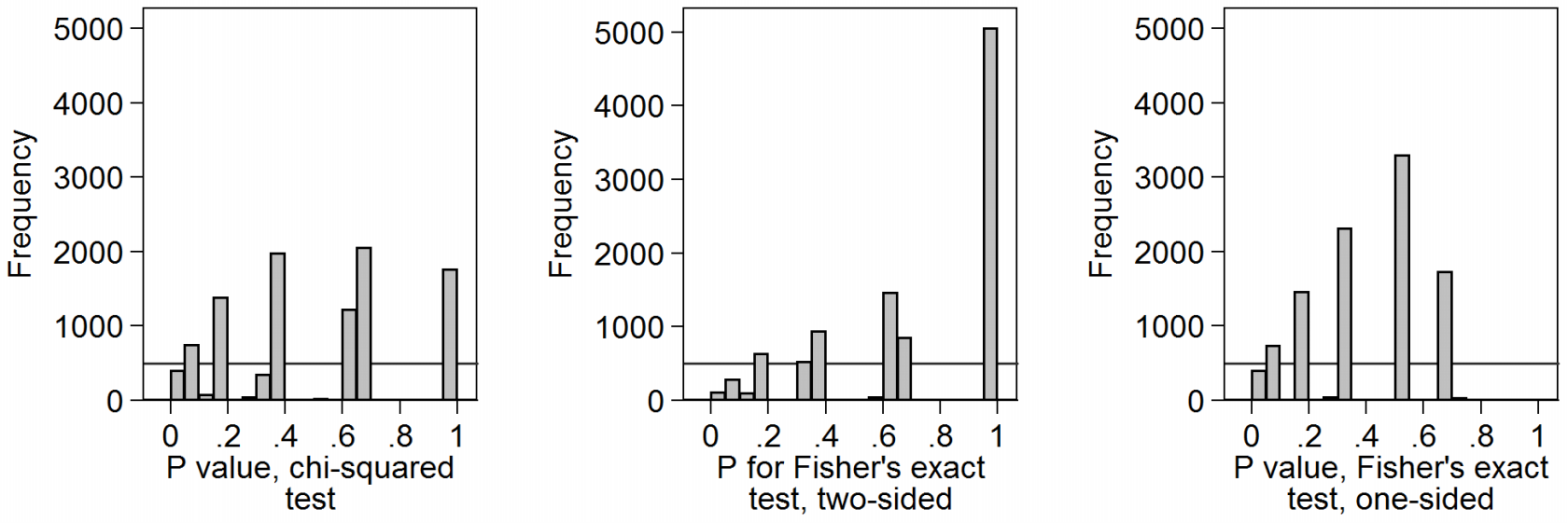
Distribution of P-values for chi-squared tests and Fisher’s exact tests for two by two tables. Chi-squared and Fisher’s exact test, both two-sided and one-sided, were calculated for the comparison of two samples of size 10 with a binary outcome variable with probability 0.5 of being 0 and 0.5 of being 1.

Figure 4 shows the results of an uncorrected chi-squared test and Fisher’s exact test, both two-sided and one-sided, for 10,000 independent comparisons of two samples of size 10 with a binary outcome variable with probability 0.5 of being 0 and probability 0.5 of being 1. The uncorrected chi-squared test and Fisher’s exact tests were carried out. For small samples like two groups of size 10 a chi-squared test would not be valid, because most such tables will have an expected frequency less than 5.0. Fisher’s exact test might be preferable. However, both clearly show a distribution very far from Uniform, with only a few P-values being represented.

Because the number of two by two tables with two row totals equal to 10 is limited, there are only 31 possible values for the chi-squared statistic in this simulation, five of the 36 possible tables having chi-squared  =  0.0 and there being one table for which chi-squared is undefined. Because P is recorded to only 3 decimal places, there are even fewer possible values for the probability. Hence the Uniform distribution for P-values should not be valid for chi-squared tests in small samples and it is not. It is not valid for Fisher’s exact either, for the same reason.

### 5. Chi-squared and Fisher’s exact test for large samples

**Figure 5:**
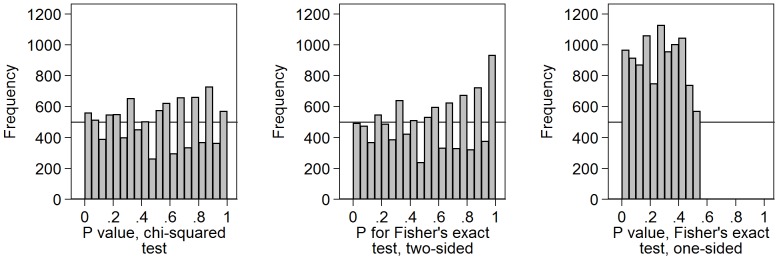
P-values for chi-squared and Fisher’s exact tests for large samples with fixed size groups. Chi-squared and Fisher’s exact test, both two-sided and one-sided, were calculated for10,000 comparisons of two samples of size 1,000 with a binary outcome variable with probability 0.5 of being 0 and 0.5 of being 1.

Figure 5 shows the results of an uncorrected chi-squared test and Fisher’s exact test, both two-sided and one-sided, for 10,000 comparisons of two samples of size 1,000 with the same binary outcome variable. The two sided probabilities for Fisher’s exact test and the chi-squared test will be approximately the same for a large sample. Despite the size of the sample and the large number of possible tables, the distribution shows pronounced peaks and troughs. It does not conform closely to a Uniform distribution, as does Figure 1, for example. Another run gave the peaks and troughs in the same places. This applied to both the chi-squared test and to Fisher’s exact test.

I considered Fisher’s exact test one-sided. This is also testing a null hypothesis which is known to be true. This might be expected again to lead to a Uniform distribution. However, here the one-sided test Fisher’s exact test as implemented in Stata 12 sums all probabilities smaller than that for the table observed for tables in the direction away from the expected frequencies. The direction is not specified in advance, so the resulting P-values are never much greater than 0.5. It is not a true one-sided test. Here the largest P-value was 0.513.

**Figure 6:**
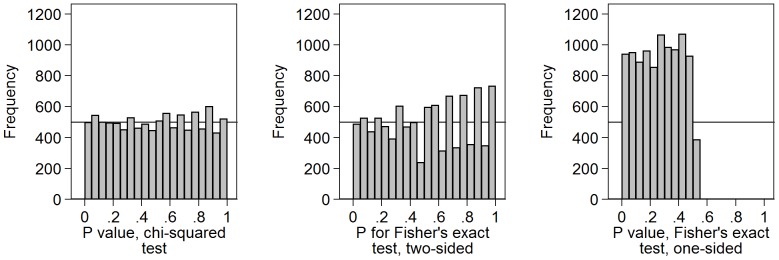
P-values for chi-squared and Fisher’s exact tests for large groups of varying size. Chi-squared and Fisher’s exact test, both two-sided and one-sided, were calculated for10,000 comparisons in samples of size 2,000, both group and outcome having probability 0.5 of being 0 and 0.5 of being 1.

I thought it possible that the peaks and troughs were the result of having one fixed margin, which would correspond to the fixed treatment group sizes is a randomised trial. I therefore repeated the simulation, generating groups in the same way as the binary outcome, so that neither row nor column totals were fixed. The resulting distributions (Figure 6) exhibited peaks and troughs in the same places, though these were not so large as for fixed size groups.

## Discussion

I have not found the proposition of a Uniform distribution for P-values used in any published paper, but I have come across it twice recently in proposals for analysis. I thought it would be worthwhile to point out its deficiency before it started to be used and copied.

As expected, the Uniform distribution for P-values worked well for independent two sample t tests comparing data from a Normal distribution. It did not work well for data from a skewed distribution, where the test of significance was not valid. This lack of validity is shown by the low number of P-values less than 0.05. In Figure 2, the proportion is 3.7% rather than the 5% expected for a valid test, whereas in Figure 1, where the test is valid, it is 4.8%. It did not work well for correlated two sample t tests, either. Both of these findings confirmed my prior ideas about this method. The finding for non-Normal data is particularly relevant to the idea that a reviewer could look at tests comparing mean age between randomised groups over several studies. Age seldom follows a Normal distribution in any sample of people.

More surprising was the lack of fit to the Uniform distribution found for P-values for chi-squared tests based on a large sample. These tests should be valid, because the expected frequencies are close to 500, far greater than the 5.0 required by the usual criterion. The proportion with P<0.05 is 5.6%, close to the 5% expected for a valid test when all null hypotheses are true. I found the graininess and unevenness of the distribution of P-values for chi-squared tests, even with a very large sample, quite surprising.

There is a large literature criticising the use of significance tests of differences in baseline variables in randomised clinical trials [1], [2], [3], [4], [5], [6]. The usual argument is that, if groups are chosen randomly, the two groups are from the same population and any null hypothesis of a zero difference between their populations is true. Testing is superfluous, because any significant difference is a type I error, one of the 5% expected to occur. Even very small P values may happen occasionally when the null hypothesis is true. Not all agree and Berger [7] argues that such tests are a valid method to identify possible bias and subversion in allocation. Subversion can and does occur; Kennedy and Grant [8] reported a trial where some centres used a central randomisation system and others used sealed envelopes held locally. There was a significant difference in age between randomised groups in the local allocation centres, for three clinicians in particular, but not for the central allocation centres. Berger and Exner [9] present a test for imbalance which can be more powerful than baseline comparisons, being based on response data rather than baseline.

Most triallists who present such tests, about half in the study by Pocock *et al.* [6], do not do so because they suspect that their own trial has been subverted. They do it because they wish to reassure themselves and their readers that chance has not led to an imbalance for which they need to adjust, or to identify variables for which adjustment should be made. But this comparison is irrelevant to the decision to adjust for baseline variables. If a baseline variable is prognostic for the outcome, researchers should adjust for it whether the groups differ or not, as adjustment will improve the estimate of the treatment effect. If a baseline variable is not prognostic for the outcome, researchers do not need to adjust for it even when the groups differ, as adjustment will not improve the estimate of the treatment effect. The triallist should adjust for known prognostic variables and should be able to plan the adjustment before a patient has been recruited.

For many years, researchers reporting clinical trials have been urged to replace P values with estimates and confidence intervals [10], [11]. One possible way forward might be to acknowledge that some imbalance is inevitable and, rather than ask whether there is evidence of it, try to quantify it. Every trial could report an imbalance coefficient. How this might be done is beyond the scope of this article, but one way forward might be to combine as many baseline variables as possible, using principal components analysis, standardise the first principal component, and find the difference for this between groups. Much work would be needed on the implications of this idea and the robustness of the coefficient before it could be adopted.

These simulations show that this idea of a Uniform distribution for P-values testing true null hypotheses just will not work under many circumstances. It is unlikely to work in most applications and wrong conclusions may be drawn. For example, in their review of published trials, Schulz et al. [1] interpreted the 2% of baseline comparisons being statistically significant, significantly lower than the expected rate of 5%, as plausibly being due to a few investigators having decided not to report statistically significant comparisons, in the belief that this would enhance the credibility of their trials. Some of these tests may be invalid and they are certainly not all independent. As Figures 2 and 3 show, it is also plausible that fewer than 5% of tests might be found to be significant as a result of this, even though all the tests were reported and there was no concealment on the part of the triallists.
